# Supplementary material for: Predictors of incident viral symptoms ascertained in the era of COVID-19
Source: PLoS One. 2021 Jun 17;16(6):e0253120. doi: 10.1371/journal.pone.0253120 (PMC8211176; doi:10.1371/journal.pone.0253120)
Supplement: S1 Table — (DOCX) [file pone.0253120.s001.docx]

| **Number of weeks of follow-up** | **Percentage that completed at least one daily survey per week** | **Percentage that completed the weekly survey** |
| --- | --- | --- |
| 1 | 100% | 96% |
| 2 | 96% | 66% |
| 3 | 95% | 67% |
| 4 | 97% | 59% |
| 5 | 99% | 21% |
| 6 | 100% | 89% |

**S1 Table. Proportion of participants completing at least one daily survey per week and the proportion completing weekly surveys.**
