# Supplementary material for: Predictors of incident viral symptoms ascertained in the era of COVID-19
Source: PLoS One. 2021 Jun 17;16(6):e0253120. doi: 10.1371/journal.pone.0253120 (PMC8211176; doi:10.1371/journal.pone.0253120)
Supplement: S2 Table — (DOCX) [file pone.0253120.s002.docx]

| **Symptom** | **Prevalence (Total N=424)** |
| --- | --- |
| Median number of other symptoms (IQR) | 2.0 (1.0-3.0) |
| Scratchy throat | 161 (38.0%) |
| Painful throat | 64 (15.1%) |
| Cough | 106 (25.0%) |
| Runny nose | 150 (35.4%) |
| Temperature | 36 (8.5%) |
| Muscle ache | 152 (35.8%) |
| Nausea, vomiting, diarrhea | 131 (30.9%) |
| Shortness of breath | 64 (15.1%) |
| Lost taste or smell | 15 (3.5%) |
| Red eyes | 30 (7.1%) |

**S2 Table. Specific symptoms reported in addition to fevers and chills**
