# Supplementary material for: Predictors of incident viral symptoms ascertained in the era of COVID-19
Source: PLoS One. 2021 Jun 17;16(6):e0253120. doi: 10.1371/journal.pone.0253120 (PMC8211176; doi:10.1371/journal.pone.0253120)
Supplement: S3 Table — Derived backwards stepwise elimination of covariates (see Methods). * overall heterogeneity. † heterogeneity of non-reference levels. # linear trend. (DOCX) [file pone.0253120.s003.docx]

| **Characteristic** | **Odds ratio** | **95% CI** | **p-value** | **Group p-value** |
| --- | --- | --- | --- | --- |
| **Age category** |  |  |  |  |
| 18-29 | reference |  |  | 0.019* |
| 30-39 | 0.82 | 0.56, 1.19 | 0.29 | 0.021† |
| 40-49 | 0.89 | 0.61, 1.30 | 0.55 | 0.007# |
| 50-59 | 0.85 | 0.57, 1.27 | 0.43 |  |
| 60+ | 0.49 | 0.32, 0.77 | 0.002 |  |
| **Race/ethnicity** |  |  |  |  |
| White | reference |  |  | 0.43* |
| Black | 0.98 | 0.31, 3.06 | 0.97 | 0.29† |
| Hispanic (any race) | 1.06 | 0.72, 1.55 | 0.78 | 0.84# |
| Asian or Pacific Islander | 0.66 | 0.41, 1.08 | 0.10 |  |
| Other (including multiracial) | 1.33 | 0.71, 2.51 | 0.38 |  |
| **Female Biological Sex** | 1.82 | 1.39, 2.39 | <0.001 |  |
| **MacArthur Subjective Social Status Ladder** | 0.87 | 0.81, 0.94 | <0.001 |  |
| **High blood pressure** | 1.40 | 1.05, 1.85 | 0.02 |  |
| **At least weekly exercise** | 0.68 | 0.53, 0.89 | 0.004 |  |
| **Cigarettes: any use in last 30 days** | 1.73 | 1.14, 2.61 | 0.01 |  |
| **Sanitized phone** | 0.70 | 0.53, 0.92 | 0.012 |  |
| **Any household symptoms, 6-12 days ago** | 2.08 | 1.61, 2.70 | <0.001 |  |
| **Maximum contacts (per 10), 6-12 days ago** | 4.79 | 1.90, 12.10 | <0.001 |  |
| **Calendar date (linear)** | 0.94 | 0.90, 0.98 | 0.005 |  |
| **Calendar date (non-linear)** | 1.05 | 1.00, 1.10 | 0.054 |  |

**Table S3. Sensitivity Analysis of Independent Predictors of Incident Symptoms Excluding Anemia.**

Derived backwards stepwise elimination of covariates (see methods).

  * overall heterogeneity

  † heterogeneity of non-reference levels

  # linear trend
