# Supplementary material for: Predictors of incident viral symptoms ascertained in the era of COVID-19
Source: PLoS One. 2021 Jun 17;16(6):e0253120. doi: 10.1371/journal.pone.0253120 (PMC8211176; doi:10.1371/journal.pone.0253120)
Supplement: S4 Table — Derived backwards stepwise elimination of covariates (see Methods). * overall heterogeneity. † heterogeneity of non-reference levels. # linear trend. (DOCX) [file pone.0253120.s004.docx]

| **Characteristic** | **Odds ratio** | **95% CI** | **p-value** | **Group p-value** |
| --- | --- | --- | --- | --- |
| **Age category** |  |  |  |  |
| 18-29 | reference |  |  | 0.22* |
| 30-39 | 0.83 | 0.60, 1.16 | 0.28 | 0.221† |
| 40-49 | 0.87 | 0.62, 1.21 | 0.41 | 0.14# |
| 50-59 | 0.95 | 0.68, 1.33 | 0.77 |  |
| 60+ | 0.69 | 0.48, 0.99 | 0.046 |  |
| **Race/ethnicity** |  |  |  |  |
| White | reference |  |  | 0.53* |
| Black | 1.05 | 0.46, 2.39 | 0.90 | 0.30† |
| Hispanic (any race) | 1.09 | 0.80, 1.49 | 0.57 | 0.79# |
| Asian or Pacific Islander | 0.74 | 0.49, 1.13 | 0.16 |  |
| Other (including multiracial) | 1.31 | 0.79, 2.16 | 0.29 |  |
| **Female Biological Sex** | 1.74 | 1.38, 2.18 | <0.001 |  |
| **MacArthur Subjective Social Status Ladder** | 0.89 | 0.83, 0.94 | <0.001 |  |
| **Anemia** | 1.41 | 1.13, 1.76 | 0.002 |  |
| **Congestive Heart Failure** | 1.74 | 1.12, 2.71 | 0.014 |  |
| **Asthma** | 1.37 | 1.07, 1.76 | 0.012 |  |
| **High blood pressure** | 1.30 | 1.04, 1.63 | 0.019 |  |
| **At least weekly exercise** | 0.58 | 0.47, 0.71 | <0.001 |  |
| **Cigarettes: any use in last 30 days** | 1.81 | 1.32, 2.50 | <0.001 |  |
| **Sanitized phone** | 0.79 | 0.63, 0.99 | 0.038 |  |
| **Any household symptoms, 6-12 days ago** | 2.06 | 1.67, 2.55 | <0.001 |  |
| **Maximum contacts (per 10), 6-12 days ago** | 4.42 | 1.95, 10.00 | <0.001 |  |
| **Calendar date (linear)** | 0.94 | 0.90, 0.97 | <0.001 |  |
| **Calendar date (non-linear)** | 1.05 | 1.01, 1.09 | 0.02 |  |

**Table S4. Sensitivity Analysis of Independent Predictors of Incident Symptoms Including Participants with Atrial Fibrillation, Coronary Artery Disease, Congestive Heart Failure, Chronic Obstructive Pulmonary Disease, and Asthma.**

Derived backwards stepwise elimination of covariates (see methods).

  * overall heterogeneity

  † heterogeneity of non-reference levels

  # linear trend
