# Supplementary material for: Predictors of incident viral symptoms ascertained in the era of COVID-19
Source: PLoS One. 2021 Jun 17;16(6):e0253120. doi: 10.1371/journal.pone.0253120 (PMC8211176; doi:10.1371/journal.pone.0253120)
Supplement: S1 File — (PDF) [file pone.0253120.s005.pdf]

Sections

Baseline Data Collection

Baseline Survey

Baseline Data Collection

In what country is your primary residence?

What is the ZIP code (if in the U.S.) or postal code of your primary residence?

Have you been tested for the novel coronavirus (the virus that causes COVID-19)?

- Yes
- No
- Other

What was the result?

- Positive
- Negative
- I don't know yet
- Other

How many weeks ago did you test positive (put 0 if this week)?

Which of the following describes your primary area of employment?

- Healthcare
- Education
- Retail
- Transportation
- Arts, entertainment, and recreation
- Hospitality and food services

- Finance and insurance
- Scientific and technical services
- Utilities
- Construction
- Manufacturing
- Other

Are you aware of any novel coronavirus (the virus causing COVID-19) infected individuals in your COUNTY (or local area equivalent if your area does not have counties)?

- Yes
- No

How worried are you that the health of you or your loved ones will be affected by the novel coronavirus (the virus causing COVID-19)?

- Extremely worried
- Very worried
- Somewhat worried
- A little worried
- Not worried at all

Has your local government issued or continued any of the following restrictions? (CHECK ALL THAT APPLY)

- [ ] School closures
- [ ] Restricted gatherings at (or closed) bars, restaurants, and/or theaters

- [ ] Restricted gatherings of a certain number of individuals
- [ ] Recommended working from home or not working
- [ ] Shelter in place (required to stay home except for essential activities)
- [ ] Other restrictions

How have your hand hygiene practices (washing hands and/or using hand sanitizer) changed since learning about the novel coronavirus (the virus causing COVID-19)?

- I wash or sanitize my hands MUCH MORE frequently than before
- I wash or sanitize my hands SOMEWHAT MORE frequently than before
- I wash or sanitize my hands A LITTLE MORE frequently than before
- I have not made any changes
- I wash or sanitize my hands A LITTLE LESS frequently than before
- I wash or sanitize my hands SOMEWHAT LESS frequently than before
- I wash or sanitize my hands MUCH LESS frequently than before

Have you sanitized your mobile phone (such as by using sanitizing wipes or hand sanitizer) since learning of the novel coronavirus (the virus causing COVID-19)?

- Yes
- No
-

Other

Do any school-aged (K-12 or equivalent) children live with you?

Yes

No

Other

Do you have a college-aged child (under the age of 25) who usually does not live in your home but who has returned home and is living in your house because of the coronavirus pandemic?

Yes

No

What date did they return? (Your best guess is fine.)

MM/DD/YYYY

What school were they attending?

School

Where is the school located?

Do you live with or have continued regular in-person contact with an elderly person (over 65 years of age) or someone susceptible to illness (being immunocompromised or having a pre-existing medical condition)?

Yes

No

Other

Do you have any pets at home?

Yes

No

Other

What pets live with you (CHECK ALL THAT APPLY):

Dog(s)

Cat(s)

Bird(s)

Reptile(s)

Other

Did you have a flu shot (influenza vaccine) in the past year?

Yes

No

Other

Have you had cold or flu symptoms (enough that you would say that you had a cold or the flu) in the past year?

Yes

No

How many cold or flu illnesses in the past year were associated with a fever

(Temperature > 101.3 F or > 38.5 C)?

- None
- 1-3
- 4-6
- More than 6

When was the last one?

weeks ago

How many cold or flu illnesses in the past year were NOT associated with a fever (Temperature > 101.3 F or > 38.5 C)?

- None
- 1-3
- 4-6
- More than 6

When was the last one?

weeks ago

On average, how often have you exercised (enough to breathe heavily and/or sweat) over the past year?

- Never or rarely
- Less than once a month
- More than once a month but less than once a week
- About once a week
- More than once a week but less than 4 times a week
- More than 4 times a week
- Other

Click here to finish

Sections

Baseline Data Collection

Demographics Survey

Baseline Data Collection

Think of this ladder as representing where people stand in your country. At the top of the ladder are the people who are the best off -- those who have the most money, the most education and the most respectful jobs. At the bottom are the people who are the worst off -- who have the least money, least education, and least respectful jobs or no job. The higher up you are on the ladder, the closer you are to the people at the very top; the lower you are, the closer you are to the people at the very bottom.

Where would you place yourself on this ladder?

What is the highest level of education you have achieved?

- No formal schooling
- Some school, but did not graduate high school
- High school diploma or equivalency (e.g., GED)
- Associate degree (e.g., junior college)
- Some college, but did not graduate college
- Bachelor's degree
- Master's degree
- Doctorate (PhD)
- Professional doctorate (MD, JD, DDS, etc.)
- Other
- Don't know
- Prefer not to state

What is your biological sex?

- Male
- Female
- Prefer not to say

How would you describe your current gender identity?

- Male
- Female
- Transgender Woman (Male-to-Female)
- Transgender Man (Female-to-Male)
- Genderqueer
- Another Gender Identity
- Decline to state

What gender identity do you identify with? (Optional)

What is your racial background? CHECK ALL THAT APPLY.

- [ ] Black or African American
- [ ] White
- [ ] Asian (including South Asian and Asian Indian)
- [ ] Native Hawaiian or Pacific Islander
- [ ] American Indian or Alaska Native
- [ ] Some other race
- [ ] Don't know

What is your Asian background?

- Chinese
- Filipino
- Asian Indian
- Japanese
- Korean
- Vietnamese
- Other Asian or Mix

What is your Pacific Island background?

- Native Hawaiian
- Samoan
- Guamanian or Chamorro
- Other Pacific Islander or Mix

This is a question about ethnicity, rather than race, as used in the US Census. For example, someone may be of white race and Hispanic ethnicity or black race and Hispanic ethnicity.

Are you of Hispanic, Latino or Spanish origin or ancestry?

- No
- Yes: Mexican, Mexican American or Chicano
- Yes: Puerto Rican
- Yes: Cuban
- Yes: Other or Mixed Hispanic, Latino or Spanish origin
- Don't know
- Prefer not to state

Click here to finish

Your Smoking History

Sections

Baseline Data Collection

Baseline Data Collection

Have you ever smoked a cigarette, even one or two puffs?

- Yes
- No
- Don't know
- Refuse to answer

Have you smoked cigarettes in the past 30 days?

- ○ Yes
- ○ No
- ○ Refuse to answer

About how many days have you smoked a cigarette in the past 30 days?

[ ]

On average, how many cigarettes per day have you smoked in the past 30 days (use 1 if less than one)

[ ] cigarettes per day

Have you ever smoked a cigar, cigarillo, or tabacco product other than cigarette, even one or two puffs?

- ○ Yes
- ○ No
- ○ Don't know
- ○ Refuse to answer

Have you smoked a cigar, cigarillo, or tobacco product other than a cigarette in the past 30 days?

○ ○

Yes

No

Don't know

Refuse to answer

About how many days have you smoked a cigar, cigarillo, or tobacco product other than cigarette in the past 30 days?

days

On average, how many cigar, cigarillo, or tobacco product (other than cigarettes) per day have you smoked in the past 30 days (use 1 if less than one)?

Have you ever used an electronic nicotine product (e-cigarette, vape nicotine), even one or two puffs?

Yes

No

Don't know

Refuse to answer

Have you used an electronic nicotine product in the past 30 days?

Yes

No

Don't know

Refuse to answer

About how many days did you use it in the past 30 days?

days

How many puffs from an e-cigarette do you typically take over the past 30

days?

How much did you spend on electronic delivery products in the past 30 days?

Dollars

Have you smoked or vaped marijuana, even one or two puffs?

- Yes
- No
- Don't know
- Refuse to answer

Have you smoked or vaped in the past 30 days?

- Yes
- No
- Don't know
- Refuse to answer

How many days did you smoke or vape in the past 30 days?

Days

Click here to finish

Daily COVID-19 Citizen Science Survey

Sections

Daily Surveys

Daily Surveys

IN THE PAST 24 HOURS: have YOU had any of the following (CHECK ALL THAT APPLY):

- [ ] A scratchy throat
- [ ] A painful sore throat
- [ ] A cough (worse than usual if you have a baseline cough)
- [ ] A runny nose
- [ ] Symptoms of fever or chills
- [ ] A temperature greater than 100.4 °F or 38.0 °C
- [ ] Muscle aches (worse than usual if you have baseline muscle aches)
- [ ] Nausea, vomiting or diarrhea
- [ ] Shortness of breath
- [ ] Unable to taste or smell
- [ ] None of the above

Did you seek medical care for these symptoms?

- ( ) Yes
- ( ) No

IN THE PAST 24 HOURS, has ANYONE (other than you) in your household had ANY of those symptoms? (scratchy/sore throat, cough, runny nose, fevers/chills/high temperature, muscle aches, nausea/vomiting/diarrhea, shortness of breath, unable to taste or smell)

- [ ] Yes
- [ ] No
- [ ] Not sure

IN THE PAST 24 HOURS, approximately how many people outside of your household did you interact with or come within 6 feet of?

Click here to finish

Weekly COVID-19 Citizen Science Survey

Sections

Weekly Surveys

Weekly Surveys

In the past WEEK, have you been tested for the novel coronavirus (the virus that causes COVID-19)?

- Yes
- No
- Other

What was the result?

- Positive
- Negative
- I don't know yet
- Other

After initiating contact with a healthcare professional, how long did you wait, in days, until you were tested? Put 0 if you were tested in less than 1 day.

day(s)

How long, after being tested, did you get your results back? Put 0 if you got your results in less than 1 day.

day(s)

Over the past WEEK, have you become aware of any novel coronavirus (the virus causing COVID-19)-infected individuals in your COUNTY (or local area equivalent if your area does not have counties)?

- Yes
- No

Over the past WEEK, how worried have you been that the health of you or

your loved ones will be affected by the novel coronavirus (the virus causing COVID-19)?

- Extremely worried
- Very worried
- Somewhat worried
- A little worried
- Not worried at all

Over the past WEEK, on average, how often have you washed or sanitized your hands?

- More than 10 times per day
- 5-10 times per day
- 2-4 times per day
- About once per day
- Less than once per day

Over the past WEEK, how many times have you visited a gym?

Over the past WEEK, how many times have you visited a restaurant (not for takeout)?

Over the past WEEK, how many times have you visited a bar?

Over the past WEEK, how many times have you visited a movie theater?

Over the past WEEK, how many times have you visited an event with more than 10 people?

Over the past WEEK, how often have you exercised for more than 20 minutes (enough to breathe heavily and/or sweat)?

[ ]

Over the past WEEK, has your local government issued or continued any of the following restrictions? (CHECK ALL THAT APPLY)

- [ ] School closures
- [ ] Restricted gatherings at (or closed) bars, restaurants, and/or theaters
- [ ] Restricted gatherings of a certain number of individuals
- [ ] Recommended working from home or not working
- [ ] Shelter in place (required to stay home except essential activities)
- [ ] Other restrictions

Over the past WEEK, on average, how many hours did you sleep per night?

[ ] hours per night

Click here to finish

Sections

Monthly Surveys

Monthly COVID-19 Citizen Science Survey

Monthly Surveys

Please answer the following for the period of the past 30 days

What best describes your current main daily activities and/or responsibilities over the past 30 days?

- Working full time
- Working part-time
- Unemployed, laid off, or looking for work
- In school (full- or part-time student)
- Stay-at-home parent or keeping household
- Retired
- Disabled
- Prefer not to state

How much of your working time is currently performed at home?

- 100% of the time
- 75-99% of the time
- 50-74% of the time
- 25-49% of the time
- 1-24% of the time
- None

Has your income changed in the past 30 days?

- Yes, it has increased
- Yes it has declined

- No, it is about the same
- Prefer not to state

In the past 30 days, by what percentage has your income increased?

[ ] %

In the past 30 days, by what percentage has your income declined?

[ ] %

In the past 30 days, have you been unemployed?

- Yes
- No
- Prefer not to state

How hard is it for you (and your family) to pay for the very basics like food, rent or mortgage, heating, etc over the past 30 days?

- Very hard
- Hard
- Somewhat hard
- Not very hard
- Don’t know
- Prefer not to state

Did you have difficulty making ends meet over the past 30 days?

- Frequently
- Occasionally
- Hardly ever
- Never
- Don’t know
- Prefer not to state

Click here to finish

Sections

Monthly Surveys

Anxiety Survey

Monthly Surveys

Over the last two weeks, how often have you been bothered by the following problems?

Feeling nervous, anxious, or on edge.

Not at all

Several days

More than half the days

Nearly every day

Not being able to stop or control worrying.

Not at all

Several days

More than half the days

Nearly every day

Worrying too much about different things.

Not at all

Several days

More than half the days

Nearly every day

Trouble relaxing.

Not at all

Several days

More than half the days

Nearly every day

Being so restless that it is hard to sit still.

Not at all

Several days

More than half the days

Nearly every day

Becoming easily annoyed or irritable.

Not at all

Several days

More than half the days

Nearly every day

Feeling afraid as if something awful might happen.

Not at all

Several days

More than half the days

Nearly every day

Click here to finish

Sections

Monthly Surveys

Mood Survey

Monthly Surveys

Over the last 2 weeks, how often have you been bothered by any of the following problems?

Little interest or pleasure in doing things.

Not at all | Several days
More than half the days | Nearly every day

Feeling down, depressed, or hopeless.

Not at all | Several days
More than half the days | Nearly every day

Trouble falling or staying asleep, or sleeping too much.

Not at all | Several days
More than half the days | Nearly every day

Feeling tired or having little energy.

Not at all | Several days
More than half the days | Nearly every day

Poor appetite or overeating.

Not at all
Several days
More than half the days
Nearly every day

Feeling bad about yourself - or that you are a failure or have let yourself or your family down.

Not at all
Several days
More than half the days
Nearly every day

Trouble concentrating on things, such as reading the newspaper or watching television.

Not at all
Several days
More than half the days
Nearly every day

Moving or speaking so slowly that other people could have noticed. Or the opposite - being so fidgety or restless that you have been moving around a lot more than usual.

Not at all
Several days
More than half the days
Nearly every day

Click here to finish
